# Adaptive Evolution of *Escherichia coli* to an α-Peptide/β-Peptoid Peptidomimetic Induces Stable Resistance

**DOI:** 10.1371/journal.pone.0073620

**Published:** 2013-09-05

**Authors:** Line Hein-Kristensen, Henrik Franzyk, Anne Holch, Lone Gram

**Affiliations:** 1 Division of Industrial Food Research, National Food Institute, Technical University of Denmark, Kgs. Lyngby, Denmark; 2 Department of Drug Design and Pharmacology, Faculty of Health and Medical Sciences, University of Copenhagen, København Ø, Denmark; 3 Department of Systems Biology, Technical University of Denmark, Kgs. Lyngby, Denmark; University of Birmingham, United Kingdom

## Abstract

Antimicrobial peptides (AMPs) and synthetic analogues thereof target conserved structures of bacterial cell envelopes and hence, development of resistance has been considered an unlikely event. However, recently bacterial resistance to AMPs has been observed, and the aim of the present study was to determine whether bacterial resistance may also evolve against synthetic AMP analogues, e.g. α-peptide/β-peptoid peptidomimetics. *E. coli* ATCC 25922 was exposed to increasing concentrations of a peptidomimetic (10 lineages), polymyxin B (10 lineages), or MilliQ water (4 lineages) in a re-inoculation culturing setup covering approx. 500 generations. All 10 lineages exposed to the peptidomimetic adapted to 32×MIC while this occurred for 8 out of 10 of the polymyxin B-exposed lineages. All lineages exposed to 32×MIC of either the peptidomimetic or polymyxin B had a significantly increased MIC (16–32×) to the selection agent. Five transfers (∼35 generations) in unsupplemented media did not abolish resistance indicating that resistance was heritable. Single isolates from peptidomimetic-exposed lineage populations displayed MICs against the peptidomimetic from wild-type MIC to 32×MIC revealing heterogeneous populations. Resistant isolates showed no cross-resistance against a panel of membrane-active AMPs. These isolates were highly susceptible to blood plasma antibacterial activity and were killed when plasma concentrations exceeded ∼30%. Notably, MIC of the peptidomimetic against resistant isolates returned to wild-type level upon addition of 25% plasma. Whole-genome sequencing of twenty isolates from four resistant lineages revealed mutations, in murein transglycosylase D (*mltD*) and outer-membrane proteins, which were conserved within and between lineages. However, no common resistance-conferring mutation was identified. We hypothesise that alterations in cell envelope structure result in peptidomimetic resistance, and that this may occur via several distinct mechanisms. Interestingly, this type of resistance result in a concomitant high susceptibility towards plasma, and therefore the present study does not infer additional concern for peptidomimetics as future therapeutics.

## Introduction

Resistance of human bacterial pathogens to conventional antibiotics has increased drastically worldwide within the last decades [1]. This has led to an intensified search for safer alternatives for which resistance is less likely to evolve [2], [3]. These include novel natural compounds with antimicrobial activity [4], inhibitors of quorum sensing [5], [6], and antimicrobial peptides (AMPs) [7], [8]. The latter group comprises host defence molecules constituting a part of the innate immune defence in all higher organisms, where they display both direct antimicrobial activity and a broad range of immuno-modulating effects [9]–[11]. Development of resistance to AMPs is considered unlikely due to their co-evolution with bacteria, and moreover their preferred target is the “Achilleś heel” of bacterial cells i.e. their distinct envelope structure [12]. Consequently, there has been increased focus on the characterisation of natural AMPs [13], [14] and on semi-synthetic [15]–[17] and synthetic analogues [18]–[20], as well as on the development of these leads into future antibiotics against human bacterial pathogens.

Exposure of bacteria to antimicrobials typically results in development of resistance, which is either mutational or adaptive. The former is considered stable and arises after mutations or acquisition of a genetic element while the latter term describes an auto-regulated phenomenon characterised by rapid induction of resistance in the presence of the drug followed by a reversal to the sensitive phenotype when drug is absent [21]. The outer membrane of Gram-negative bacteria, which acts as a semi-permeable barrier mediating decreased sensitivity to antimicrobial compounds [22], is a well-known example of the functions of adaptive resistance. Hence, AMP-induced stress has several times been shown to activate innate two-component systems leading to modifications of the cell envelope thereby decreasing the negative charge or permeability of this barrier [23]–[27]. However, the observed decrease in susceptibility is most often lost again once the exposure to AMP is ended, and it is therefore considered an induced tolerance rather than resistance introduced via genetic alterations.

Since AMPs and their analogues mimic components of the innate immune defence in humans, development of resistance towards such compounds could potentially compromise our innate immune defence [28]. Therefore it is important to consider the risk and potential consequences of emergence of AMP-resistant strains before such compounds are to be used for systemic infections [28], [29]. Only very few studies have dealt with this problem, and in most published work, the possible AMP-induced mutation frequencies have been compared to the much higher mutation frequencies usually observed for conventional antibiotics. In general, previous studies have involved short-time evolution experiments [30], [31]. A single comprehensive study, focusing on continuous selection towards a magainin 2 analogue (pexiganan), showed that prolonged exposure to an AMP indeed may result in heritable resistance [29] despite the consensus that resistance against AMPs is unlikely to evolve. This clearly indicates that the resistance issue calls for an increased focus in AMP development programs.

**Figure 1:**
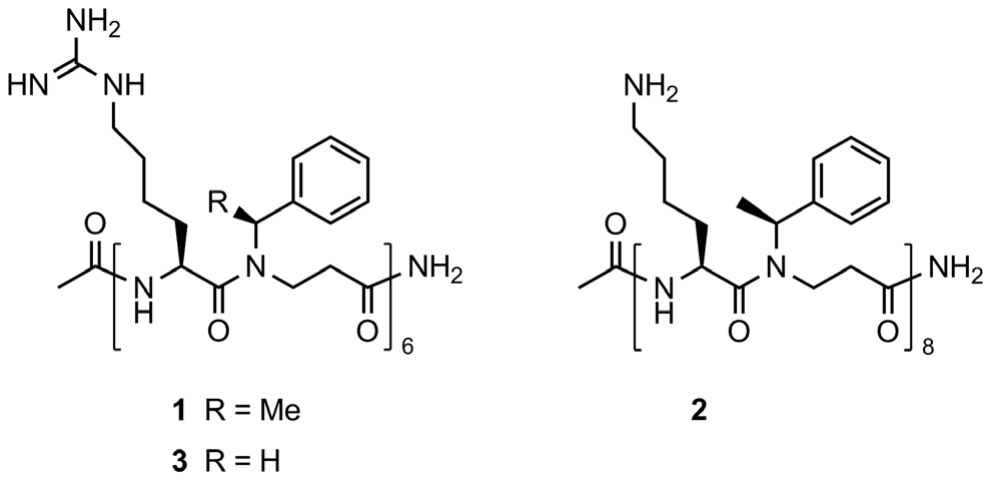
Structure of the peptidomimetics used in this study. The structure of peptidomimetic 1 used for continuous culturing of *Escherichia coli* and the structure of peptidomimetics 2 and 3 to which cross-resistance was demonstrated.

We have previously described a synthetic approach towards peptidomimetics exhibiting antimicrobial properties, and in the present study we investigate α-peptide/β-peptoid peptidomimetics (Figure 1) possessing a design with alternating N-alkylated β-alanine (β-peptoid) and α-amino acid units [32], [33]. It was demonstrated that these compounds are active against a range of nosocomial and food-borne pathogenic bacteria, and we have also shown that the chain length has a marked influence on membrane activity [34], [35]. Here, we investigate the risk of resistance development in a human pathogenic bacterium as this is a key parameter in the assessment of the therapeutic potential of AMPs and their analogues.

## Results

The aim of this study was to determine whether bacterial resistance could develop following exposure to a synthetic AMP analogue belonging to the class of antibacterial α-peptide/β-peptoid peptidomimetics. Additionally, we examined whether such resistance would hamper elimination of the bacteria by the innate immune system (exemplified by blood plasma). Also, we sought to elucidate the underlying molecular mechanisms of the peptidomimetic resistance that rapidly and consistently developed during prolonged exposure to gradually increasing concentrations of this antibacterial including polymyxin B as reference compound.

### Continuous Selection towards AMP Resistance

**Figure 2:**
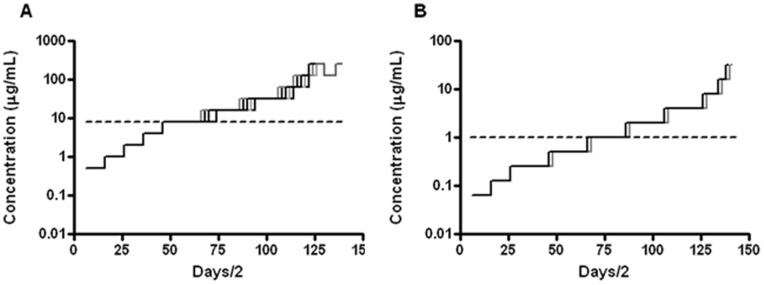
Continuous selection of *Escherichia coli*. Continuous selection exerted by peptidomimetic 1 (A), or by polymyxin B (B) leads to tolerance in *E. coli*. Solid lines represent individual lineages. All lineages exposed to the peptidomimetic were cultured up to 32×wt MIC (A); for lineages selected against polymyxin B this was eight out of ten (the last two were not able to grow after two doublings in concentration i.e. at 0.25 µg/mL). Punctuated lines indicate wt MIC of the selective agents (8 µg/mL and 1 µg/mL, respectively).

*Escherichia coli* ATCC 25922 was continuously re-cultured from a concentration of 1/16 of the wild-type (wt) MIC until adaptation to 32 times the wt MIC of either peptidomimetic 1 (i.e. 256 µg/mL) or polymyxin B (i.e. 32 µg/mL) was reached. This resistance evolved during 77 inoculations equivalent to approx. 500 generations (i.e. the culture was at each sub-culture diluted 1∶100 to a density of ∼1×10^7^ CFU/mL allowing for 6–7 generations during each of the 77 inoculations). All ten lineages selected with the peptidomimetic eventually grew at 32×wt MIC; for lineages selected with polymyxin B this was the case for eight out of ten (Figure 2). It is evident that growth was affected at an earlier stage for the polymyxin B-exposed lineages, i.e. growth was inhibited for all strains already after two doublings of the polymyxin B concentration while two lineages failed to grow further. For the peptidomimetic-exposed lineages growth was not visibly inhibited until after four doublings of the concentration of the peptidomimetic (Figure 2). Only once was the concentration decreased for a lineage supplemented with peptidomimetic (i.e. lineage 8) due to lack of growth at 32× wt MIC. After two additional passages at 16×wt MIC the lineage successfully grew at 32×wt MIC.

The four controls were transferred into fresh unsupplemented media simultaneously with the transfer of the two other groups of lineages. At the time point corresponding to 4×wt MIC of the exposed lineages (i.e. 32 µg/mL for peptidomimetic 1 and 4 µg/mL for polymyxin B) we investigated the ability of these controls to grow in the presence of the same concentration as the exposed lineages. Two controls exposed to peptidomimetic 1 at 4×wt MIC failed to grow but one of the two controls exposed to 4×wt MIC of polymyxin B became outgrown. Growth was retained for three additional passages at the same concentration of polymyxin B indicating that the spontaneous mutation rate in the presence of polymyxin B may be higher than that seen for peptidomimetic 1.

### Population MIC after Selection

MIC was determined for the selection agent against revived outgrown cultures at 32×wt MIC of all lineages in the two groups (Table 1 and 2). Only five out of ten of the freezing stocks of the peptidomimetic-exposed lineages could be re-cultured in peptidomimetic-supplemented media, though all of the eight polymyxin B-exposed freezing stocks grew well. We ascribe this to stress induced by freezing for some lineages as the cultures subsequently grew in unsupplemented substrate with retained resistance. A dramatic increase in MIC was seen for both the peptidomimetic- and the polymyxin B-exposed lineages showing that resistance had developed against the selection agent (Table 1 and 2). For the five peptidomimetic-exposed lineages, MIC against peptidomimetic 1 was increased to 128–512 µg/mL from an initial level of 8 µg/mL for the wild-type strain corresponding to a 16- to 64-fold increase. The eight polymyxin B-exposed lineages all had a MIC value of 32 µg/mL polymyxin B, i.e. a 32-fold increase in MIC as compared to the wild-type strain. The four controls only showed minor increases in MIC as compared to the wild-type strain.

**Table 1:** Minimum Inhibitory Concentration (µg/mL) of the adapted *E. coli* lineages against peptidomimetic 1 immediately after peptidomimetic adaptation and after 35 generations with no peptidomimetic.

Lineage number	MIC^a^ against peptidomimetic 1 after selection	MIC^a^ against peptidomimetic 1 after 35 generationsw/o selection
1	b	128–256
2	256	256
3	b	128
4	256	128
5	64–128	128
6	b	64–128
7	128	128
8	b	32–64
9	b	128
10	256	32
Control 1^c^	32	16–32
Control 2	8	8

a MIC values are based on two technical duplicates.

b Not determined due to lack of growth of revived freezing stocks in supplemented media.

c *E. coli* ATCC 25922 had a wt MIC of 8 µg/mL.

**Table 2:** Minimum Inhibitory Concentration (µg/mL) of the adapted *E. coli* lineages against polymyxin immediately after polymyxin adaptation and after 35 generations with no polymyxin.

Lineage number	MIC^a^ against polymyxin after selection	MIC^a^ against polymyxin after 35 generations w/o selection
1	b	b
2	32	32
3	32	32
4	32	32
5	32	32
6	32	32
7	b	b
8	32	32
9	32	32
10	32	32
Control 1^c^	8	0.5
Control 2	1	0.5–1

a MIC values are based on two technical duplicates.

b Not determined since lineage was unable to grow at 32×wt MIC.

c *E. coli* ATCC 25922 had a wt MIC of 1 µg/mL.

We determined potential cross-resistance to the other selection agent (i.e. peptidomimetic 1 vs. polymyxin B) and to an aminoglycoside antibiotic (gentamicin) and a cell wall-active antibiotic (ampicillin). No cross-resistance was found to these two conventional antibiotics for any of the lineages (not shown). Some cross-resistance was found against polymyxin B for some of the peptidomimetic-exposed lineages (2- to 16-fold increase in MIC as compared to that of the wild type, not shown), and against peptidomimetic 1 for the polymyxin B-exposed lineages (2- to 8-fold increase in MIC as compared to that of the wild type, not shown).

### Stability of Resistance

To establish whether the acquired resistance was heritable all lineages were revived in unsupplemented media and then cultured for five passages (corresponding to ∼35 generations) in the absence of the compound applied for selection. All ten lineages tolerant to the peptidomimetic were successfully revived, and again we performed MIC determinations against both selection agents for all lineages. The lineages exposed to peptidomimetic 1 displayed high levels of resistance against this compound even after growth in unsupplemented media confirming the heritability of resistance (Table 1). The ten lineages displayed MIC values against peptidomimetic 1 between 32 µg/mL (4×wt MIC) and 256 µg/mL (32×wt MIC); the lowest MIC value was seen for lineage no. 10 which showed a strong decrease as compared to the initial level of 256–512 µg/mL indicating that this lineage may be less stable than the others. Similarly, the MIC for polymyxin B against the polymyxin B-exposed lineages remained high upon culturing for five passages (equal to approx. 35 generations) in unsupplemented media (Table 2). The low level of cross-resistance seen in the two groups of lineages against peptidomimetic 1 and polymyxin B, respectively, was lost after removal of the selection pressure, and became similar to that of the controls (not shown).

### Single-culture Isolate MIC after Selection

**Figure 3:**
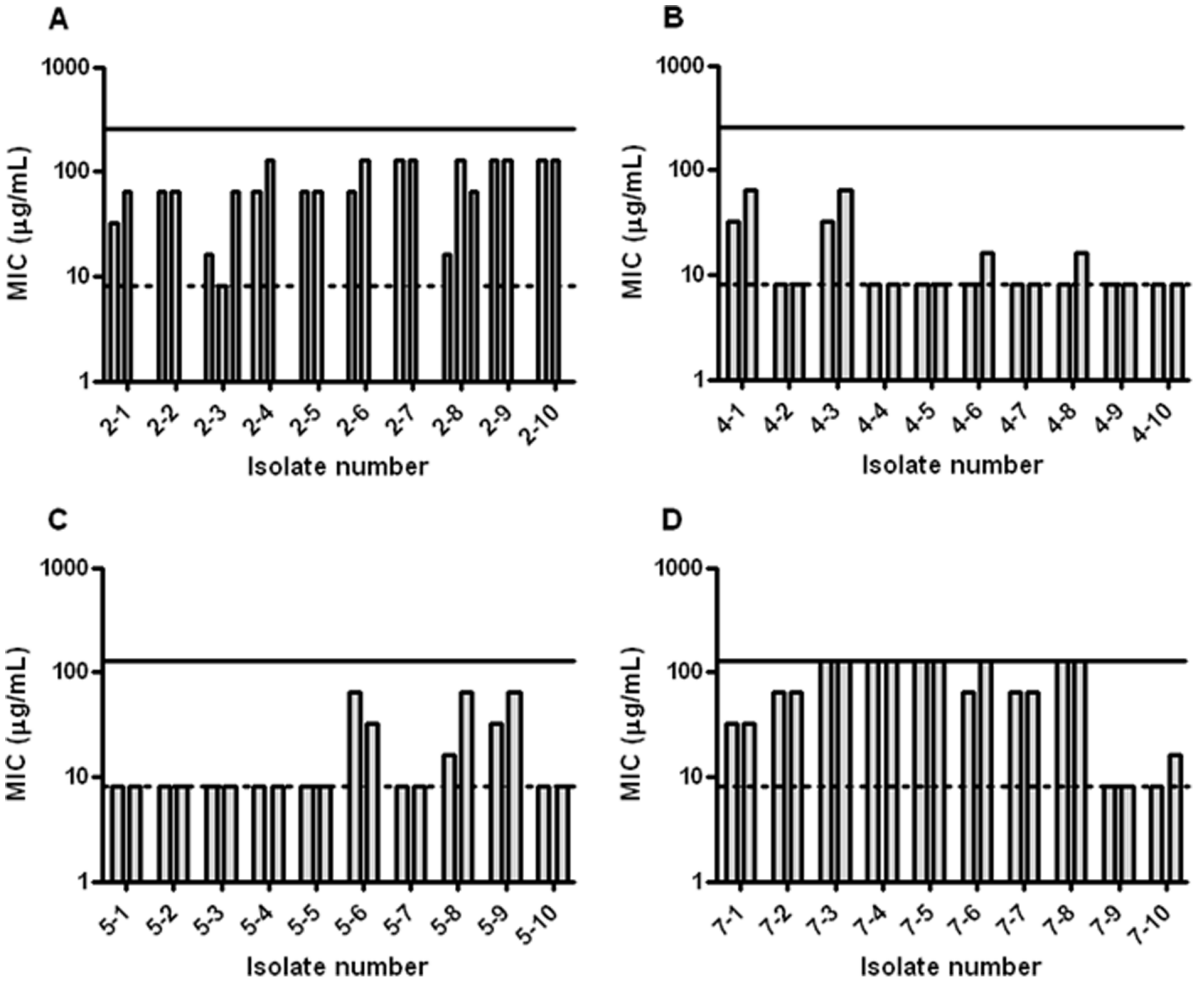
Heterogeneity in lineage populations. Minimum Inhibitory Concentration (MIC) for peptidomimetic 1 against population isolates of lineage no. 2 (A), lineage no. 4 (B), lineage no. 5 (C) and lineage no. 7 (D). Bars indicate biological replicates; MIC for all isolates was determined twice, a third replicate was performed for isolates 2–3 and 2–8 due to large variations in results. Susceptibility to peptidomimetic 1 varies widely within the populations. Solid line: population MIC; punctuated line: wt MIC (8 µg/mL).

From each of the lineages cultured at 32×wt MIC, ten individual isolates were randomly selected from plating the populations on non-selective plates. We chose to focus specifically on the isolates from four of these lineages; lineage no. 2 and no. 4 which had a population MIC of 256 µg/mL, grew well in peptidomimetic-supplemented media and grew with different colony morphologies (normal as well as larger more light colonies) and lineages no. 5 and no. 7, which had a population MIC of 128 µg/mL, grew more slowly than lineages no. 2 and no. 4 in supplemented media and appeared as homogenous colonies when plated. To this end, it seemed likely that different genetic events had occurred in these populations, and that this might be reflected in the resistance profiles of the isolates from the four populations. Hence, we determined the MIC against peptidomimetic 1 for the forty isolates from these four lineages (Figure 3). All four populations were heterogeneous displaying large differences in MIC values of individual isolates. The ten isolates from lineage no. 2 displayed MIC values within the range 8–128 µg/mL (Figure 3A). Two of the ten isolates appeared to be particularly unstable; in three biological replicates isolate 2–3 exhibited MIC values of 8, 16 and 64 µg/mL, while the MIC values for isolate 2–8 were 16, 64 and 128 µg/mL for the three biological replicates. Remarkably, from lineage no. 4, eight out of ten isolates exhibited a MIC value identical to that of the wild type (i.e. 8 µg/mL) while the other two isolates displayed a 4-fold lower MIC as compared to that of the lineage population i.e. 256 µg/mL (Figure 3B). This trend was similar to that of lineage no. 5, where seven out of ten isolates had the same MIC value as the wild type (i.e. 8 µg/mL) while none of the other three isolates displayed the same MIC as the entire population i.e. 128 µg/mL (Figure 3C). Overall, these two populations were evidently less resistant (or stable) than lineage no. 2. In contrast to this, the ten isolates from lineage no. 7, which likely reflects the diversity within the population, appeared more similar to those of lineage no. 2 (Figure 3A and D). Hence, these ten isolates displayed MIC values in the range of 8–128 µg/mL, with the highest values corresponding to the population MIC (Figure 3D). Differences in colony morphology were not reflected in a significantly altered MIC value. Additionally, individual colonies were verified as *E. coli* by 16 S rRNA sequencing as well as by a range of standard phenotypic tests (not shown). Therefore it appears that it is a general trend that the vast majority of cells have a lower MIC than the population as a whole and that a large degree of heterogeneity exists within the lineage populations.

### Cross-resistance

**Figure 4:**
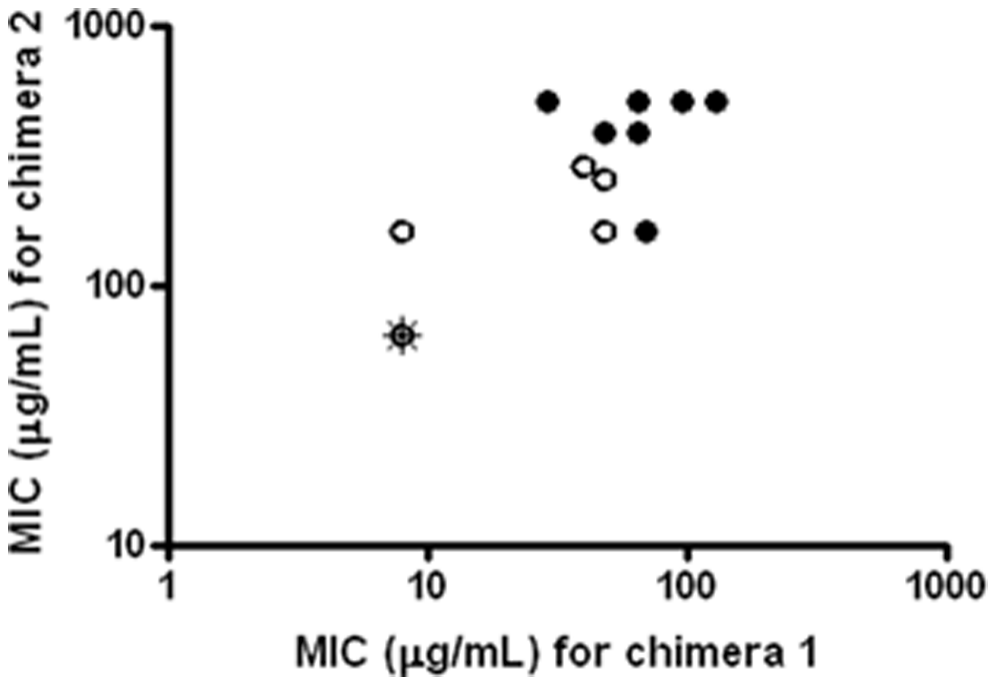
Cross-resistance between peptidomimetic 1 and 2. Association between Minimum Inhibitory Concentration (MIC) for peptidomimetic 1 and peptidomimetic 2 against lineage no. 2 (black circles) and lineage no. 5 (white circles). *E. coli* ATCC 25922 MIC is highlighted with an asterisk (i.e. MIC values of 8 µg/mL and 64 µg/mL for peptidomimetic 1 and 2, respectively). This data point is shared with six of the ten isolates from lineage no. 5. Also, for lineage 2 three isolates (2–7, 2–9 and 2–10) shared the same MIC values (peptidomimetic 1∶256 µg/mL, peptidomimetic 2∶512 µg/mL) as did the two isolates 2–4 and 2–6 (peptidomimetic 1∶96 µg/mL, peptidomimetic 2∶512 µg/mL). All values are based on two biological replicates; the mean value is displayed when results varied.

Since AMPs are almost ubiquitous in nature, the potential of cross-resistance of peptidomimetic-resistant bacteria to natural AMPs constitutes an important issue to clarify. Therefore we determined MIC values against polymyxin B (bacterial), protamine (salmon), KR-12 (a short analogue of the human cathelicidin LL-37), IsCTp (scorpion), Pep-1-K (viral) and melitin (honey bee venom) for all twenty isolates from lineages no. 2 and 5. These two lineages were chosen because significant differences in the level of resistance of individual isolates had been found between the two (Figure 3). We did not find increased MIC values for any of these as compared to the wild type (not shown). Next, the level of cross-resistance towards peptidomimetic 2 (Figure 1) was addressed. In this case, elevated MIC values were found for all isolates originating from lineages no. 2 and 5, thus reflecting a high level of resistance against this closely related peptidomimetic. Similarly, isolates with wild-type MIC towards peptidomimetic 1 from the same lineages also had considerably lower MIC values towards peptidomimetic 2 (Figure 4). Furthermore, MIC determination for another analogue sharing the same backbone design (i.e. peptidomimetic 3, Figure 1) against the three most resistant isolates from lineage no. 2 gave MIC levels that were 8 times higher than seen for the wild type strain (i.e. 4 µg/mL vs. 32 µg/mL). The evaluation of cross-resistance against different groups of antimicrobials including conventional antibiotics, natural AMPs and peptidomimetics confirmed that the developed resistance appeared to be specific for this type of peptidomimetics.

### Effect of Human Blood Plasma on Growth and Susceptibility

AMP resistance is a potential health risk due to impairment of our own immune system, and we therefore determined plasma activity against two high-level resistant isolates, one isolate with a low level of resistance and one susceptible isolate (Table 3) and compared this to the effect on the (ancestral) wild-type bacteria to see whether susceptibility to plasma was unchanged following acquisition of resistance. Consequently, both the ancestral wild-type *E. coli* and these four selected isolates were grown in a range of 25–50% plasma. While the wild type grew well and formed a visible pellet at 50% plasma concentration, only the two isolates 4–4 and 5–6, with wt MIC and a slightly increased MIC respectively, were able to do the same. The two high-level resistant isolates 2–7 and 7-3 (Figure 3) were not able to grow at plasma concentrations above 30%. Colony counts of cells recovered from wells with high plasma concentrations gave none or a very low number of surviving cells showing that high concentrations of plasma was bactericidal to the peptidomimetic-resistant isolates. When plasma was heat-treated, a conventional method for inactivation of the complement system belonging to the innate immune system [36], [37], the two high-level resistant isolates grew well in 50% plasma similar to the wild type and the isolates with no or low resistance. No changes were seen in the MIC of hydrogen peroxide or lysozyme following acquisition of peptidomimetic resistance for isolate 2–7. Lastly, MIC determination was performed against the above four isolates in 25% plasma to see whether the presence of plasma counteracted the mechanism of resistance. In 25% plasma the MIC value of peptidomimetic 1 against the wild-type *E. coli* was only marginally affected, i.e. MIC was decreased from 8 µg/mL in MHB media to 2 µg/mL (Table 3). This weak potentiation of plasma was also seen for an isolate with wt MIC, namely 4–4 for which MIC decreased from 8 µ/mL to 1–4 µg/ml in the presence of plasma, and for an isolate 5–6, with low levels of resistance where MIC decreased from 32–64 µg/mL in MHB media to 4–8 µg/mL upon addition of plasma (Table 3). In contrast a strong potentiation of plasma was seen for the two highly resistant isolates 2-7 and 7-3, where MIC against isolate 2–7 decreased from 64–128 µg/mL to 0.25-2 µg/mL in the presence of plasma and from 128 µg/mL to 1–2 µg/mL for isolate 7-3 (Table 3).

**Table 3:** MIC (µg/mL) for wild-type *E. coli* and four peptidomimetic-adapted isolates against peptidomimetic 1 in standard MHB media and in MHB media supplemented with 25% human blood plasma.

	MIC (8 µg/mL) in		
Isolate	MHB	MHB +25% plasma	Potentiation^a^
E. coli ATCC 25922	8	2	Weak
2–7	64–128	0.25–2	Strong
4–4	8	1–4	Weak
5–6	32–64	4–8	Weak
7–3	128	1–2	Strong

a Ability of plasma to interact synergistically with peptidomimetic 1.

### Fitness Cost

An important aspect of bacterial resistance towards AMPs and their analogues is to determine whether acquisition of resistance affects the growth potential of the bacterium. Thus, the growth rates of the forty isolates, obtained from lineages no. 2, 4, 5 and 7, were compared with that of the wild-type strain in the absence of peptidomimetic. The thirty isolates from lineages no. 2, 4 and 5 grew with the same growth rate as wild-type *E. coli* ATCC 25922 (p>0.05 for all). However, one isolate from lineage no. 7 (i.e. 7-3) showed a significant decrease (p<0.05) in growth rate as compared to *E. coli* ATCC 25922 and five other isolates from lineage no. 7 (i.e. 7-2, 7-4, 7-5, 7-6 and 7-8) showed a highly significant decrease (p<0.01) in growth rate relative to the wild-type strain. The growth rates of the remaining isolates from lineage no. 7 (i.e. 7-1, 7-7, 7-9 and 7-10) were not significantly different from *E. coli* ATCC 25922 (p>0.05). Next, we determined the growth rate of one representative isolate from each lineage (i.e. 2-7, 4-4, 5-6 and 7-3) in the presence of sub-inhibitory concentrations of peptidomimetic 1. As expected from their MIC values, isolates 4-4 and 5-6 were not able to grow at low inoculums at concentrations around the MIC, and hence their growth rates in supplemented media could not be calculated. Despite the high MIC values found for 2-7 and 7-3 (128 µg/mL, Figure 3) a pronounced effect on growth was seen even at very low concentrations of peptidomimetic 1. The growth rates of both isolates were significantly reduced at a peptidomimetic concentration of 8 µg/mL corresponding to wt MIC or 1/16×isolate MIC (2–7: p<0.01; 7-3: p<0.05). Remarkably, a significantly reduced growth rate was also seen for isolate 2–7 (p<0.01) at a concentration of 4 µg/mL i.e. 1/32×MIC. In comparison, a reduced growth rate was not seen for *E. coli* ATCC 25922 when it was exposed to 1/16 or 1/32×wt MIC (p>0.05).

### Whole-genome Sequencing

Twenty isolates (five from each of the four selected lineages) exposed to peptidomimetic 1 and three isolates cultured with MilliQ water as well as the ancestral wild type were whole-genome sequenced. The wild type (*E. coli* ATCC 25922) was *de novo* assembled creating 135 contigs of 5.12 MB. The 135 contigs of the ancestral strain then served as a reference for identifying SNPs and DIPs in the evolved isolates. The sequenced isolates were divided into three groups based on their susceptibility towards peptidomimetic 1: (i) isolates with wt MIC (8 µg/mL), (ii) isolates with low levels of resistance (4–8 ×wt MIC), and (iii) isolates that were highly resistant (8–16 ×wt MIC) (Table 4). The pattern of SNPs/DIPs varied between the lineages, but all isolates from lineages no. 4 and 5 had acquired the same mutations (Table 5) indicating clonality. Typically four to six mutations (resulting in amino acid changes) were detected in each isolate. Four of five isolates from lineage no. 2 and three of five isolates from lineage no. 7 displayed identical mutation patterns, albeit with difference between the two lineages. Although there were clear differences in the exact mutations between isolates from separate lineages, most (9 of 16) were present in genes/proteins related to surface structures of the bacterial cell. Only one mutation in the *gadB* gene encoding glutamate decarboxylase was found in all 20 isolates, however, this mutation was also detected in the control isolates. All three controls contained ∼100 amino acid-changing mutations indicating that without selection pressure the population will evolve rapidly. We identified three SNPs and one DIP, which only were present in six (of a total of eight) highly resistant isolates from lineages no. 2 and 7. Five of these six isolates belonged to lineage no. 2, and hence these are likely clonal. Using BLAST it was determined that the four mutations were located in genes encoding a hypothetical protein, a putative outer membrane transporter, a macrolide transporter, and the membrane-bound lytic murein transglycosylase D (mltD), respectively. No change was seen in the susceptibility of the five isolates to the macrolide erythromycin as compared to the wild type (not shown). The SNP in the *mltD* gene caused a change of amino acid 55 from proline (nonpolar) to glutamine (polar), but the effect of this on the secondary structure of the protein is unknown [38]. However, these four mutations were not found in two other isolates, 7-3 and 7-4 that also displayed high levels of resistance towards peptidomimetic 1 (i.e. MIC of 128 µg/mL). These two isolates only contained mutations that were also present in two isolates with wt MIC, 7-9 and 7–10, originating from the same lineage (Table 5). There was no pattern in the mutations in the five isolates displaying low levels of resistance to peptidomimetic 1 (4-1, 4-3, 5-6, 5-8, 5-9, Figure 3 and Table 5) that differentiated them from isolates with wt MIC.

**Table 4:** Grouping of genome-sequenced peptidomimetic-adapted isolates based on comparison to wild type (wt) MIC value (8 µg/mL).

Isolates with wt MIC	Low resistance isolates: 4–8 ×wt MIC	High resistance isolates: 8–16 ×wt MIC
4–4	4-1	2–4
4–6	4-3	2–6
4–10	5-6	2–7
5–2	5-8	2–9
5–4	5-9	2–10
7–9		7–3
7–10		7–4
		7–7

**Table 5:** Distribution of single-nucleotide polymorphisms (SNPs) and deletion-insertion polymorphism (DIPs) that caused an amino acid change and had a frequency above 80% in the peptidomimetic-adapted genome-sequenced isolates.

		Lineage 2^a^					Lineage 4					Lineage 5					Lineage 7					
Gene name	Gene product	2-4^1^	2-6^1^	2-7^1^	2-9^1^	2-10^1^	4-1^2^	4-3^2^	4-4^3^	4-6^3^	4-10^3^	5-2^3^	5-4^3^	5-6^2^	5-8^2^	5-9^2^	7-3^1^	7-4^1^	7-7^1^	7-9^3^	7-10^3^	Control
*fhuF*	Ferric ion reductase	X																				
*–*	Putative autotransporter	X	X	X	X	X													X			
*mltD*	Murein transglycosylase D	X	X	X	X	X													X			
*macB* b	Macrolide transporter	X	X	X	X	X	x	X	x	x	x	x	x	x	x	x			X			
*eutEGJ*	Ethanolamine utilization	X																				
*–*	Hypothetical protein^d^	X	X	X	X	X													X			
*gadB*	Glutamate decarboxylase^d^	X	X	X	X	X	X	X	X	X	X	X	X	X	X	X	X	X	X	X	X	X
*iucC*	Siderophore biosynthesis						X	X	X	X	X	X	X	X	X	X	X	X		X	X	
*macA* c	Macrolide transporter						X	X	X	X	X	X	X	X	X	X	x	x		X	x	
*bamA*	Outer membrane protein assembly																X	X		X	X	
*blc*	Outer membrane lipocalin																X	X		X	X	
*quuQ*	Qin prophage antitermination																X	X		X	X	
*–*	Hypothetical protein																X	X		X	X	
*yhjL*	Cellulose synthase subunit BcsC^d^																			X		

a1 isolates with high levels of resistance i.e. 8–16×wild type MIC, ^2^ isolates with intermediate levels of resistance i.e. 4–8×wt MIC; ^3^ Isolates with wild type MIC.

b Two SNPs were present in the *macB* gene: one SNP causing amino acid 319 in the protein to change from Asp to Tyr (X) and one causing amino acid 505 to change from Trp to Leu (x).

c Two SNPs were present in the *macA* gene: one SNPs causing amino acid 91 in the protein to change from Val to Gly (X) and one causing amino acid 205 to change from Val to Leu (x).

d DIP mutations; all other mutations are SNPs.

## Discussion

In this study it was demonstrated that *Escherichia coli* may develop resistance to a synthetic antimicrobial peptide analogue during prolonged continuous exposure to increasing concentrations (Table 1), and this was consistently observed in all lineages. To our knowledge this is the first demonstration of development of heritable resistance to a peptidomimetic possessing an altered backbone structure. We have previously shown that these α-peptide/β-peptoid hybrids are membrane-active [35]. For long, it was believed that development of resistance to AMPs was unlikely since these compounds target the bacterial Achilles’ heel i.e. the plasma membrane structure [12]. Although these peptidomimetics indeed target the structural features of the bacterial membrane, the investigated wild-type strain of *E. coli* were found capable of developing a mechanism of resistance that circumvents the membrane activity of the peptidomimetics. A few studies have shown that resistance may develop towards other AMPs. The most comprehensive of these studies was performed with pexiganan, an analogue of the natural AMP magainin 2, against *Pseudomonas fluorescens* and *E.coli* [29]. The authors demonstrated high levels of heritable resistance in both bacteria showing that resistance mechanisms may evolve as a result of a continuous selection pressure exerted by a single compound. Other studies have not found AMP resistance as a result of continuous selection with natural or synthetic AMPs [30], [31], [39], however, these studies were performed with sub-inhibitory concentrations of the AMP without any attempts to increase the concentration. We speculate that the development of resistance shown by Perron et.al. [29] and demonstrated in the present study may be due to the gradual two-fold increments in the concentration starting from a very low level (1/16×wt MIC) of the AMP used for selection and, hence allowing sufficient time for mutation and selection.

*E. coli* bacteria possess a large mutational reservoir for increased resistance to antibiotics [40]. The differences observed in the level of resistance and stability (Table 1) indicate that distinct mutational events may have taken place between and within the lineages. We have shown that the MIC values for the peptidomimetic varied between individual isolates from all four lineages that were selected for further investigation (Figure 3). Interestingly, we did not identify any isolates displaying the population MIC in either lineage no. 2, 4 or 5; we hypothesize that this could be due to: (i) lack of stability of the population, or (ii) presence of only small fractions of highly resistant mutants. In the development of resistance to antibiotics, bacterial charity (i.e. production of indole) has been proposed to confer protection to less resistant clones in a heterogeneous population [41]. Also, it is possible that epigenetic events such as changes in gene expression may provide temporary protection of the entire population [42].

Since previous studies had indicated moderate mutation rates for polymyxins [43], [44], we selected for polymyxin B resistance in parallel experiments. Thus, we found indications of a higher mutation rate for polymyxin B than that of peptidomimetic 1 as spontaneous growth of one of the controls at 4×wt MIC was only seen for the former (not shown). No cross-resistance was detected between peptidomimetic 1 and polymyxin B, independently of which compound had been used as the selection agent, demonstrating that different mechanisms must confer resistance against these compounds. Resistance to polymyxins has been demonstrated several times to be related to modification of the bacterial outer membrane, in particular of the lipopolysaccharide (LPS) layer [43], [45], [46]. Such modifications are most often mediated by two-component systems, which have been widely studied for their role in resistance to AMPs [23]–[25]. Two-component systems are often associated with adaptive (inducible) resistance, whereas resistance to polymyxin B in this case proved heritable in unsupplemented media., However, two-component systems e.g. PhoPQ, PmrAB and ParRS have also been shown to be sites for mutations leading to constitutive modifications of the cell surface [47]–[49]. A *pmrA* constitutive polymyxin B resistant *E. coli* mutant has previously been reported [47], and hence it is possible that two-component systems may have a role in the development of resistance in the present study, even though resistance was found to be stable.

Likewise, resistance to peptidomimetic 1 was also heritable in the examined peptidomimetic-resistant isolates. To investigate the resistance mechanism, we tested for cross-resistance to conventional antibiotics and natural AMPs. Resistance to aminoglycosides may be mediated by LPS modifications [43], but we could not demonstrate any cross-resistance to gentamicin representing this type of antibiotics. Similarly, no change was seen in the susceptibility of the isolates to a β-lactam drug (ampicillin). Importantly, we found no evidence of cross-resistance to a range of natural and semi-synthetic AMPs indicating that the resistance mechanism for membrane-active AMPs is not universal. However, pronounced cross-resistance was found to other peptidomimetics with the same scaffold (i.e. 2 and 3, Figure 1), demonstrating that: (i) replacement of homoarginine by lysine and extending the length (i.e. 12-meric 1 vs. 16-meric 2), or (ii) decreasing the degree of chirality by incorporation of achiral β-peptoid residues (i.e. peptidomimetic 3) does not preclude cross-resistance. Especially, cross-resistance to the latter peptidomimetic is interesting since the extent of chirality influences the secondary structure, which generally is believed to have a marked impact on the mechanism of action of membrane-active AMPs and peptidomimetics [50], and also have been shown to have a significant effect on the ability of these peptidomimetics to interact with human cells [51], [52].

A significant dilemma in the development and use of AMP/peptidomimetics against systemic infections is that since these compounds mimic the structure of endogenous human defence peptides, resistance to these compounds might compromise our innate immune defence [28]. We investigated this by determining whether acquisition of resistance influences the activity of other innate immune factors present in the blood. Surprisingly, the growth of the two peptidomimetic-resistant isolates 2-7 and 7-3 was hampered significantly in the presence of blood plasma. Hence, acquisition of resistance to a peptidomimetic in fact renders the bacteria more susceptible to at least the soluble components of the innate immune system present in blood plasma. We hypothesize that changes in the structure of the outer membrane confer an increased susceptibility to yet unknown plasma components that presumably interact with the bacterial membrane. Notably, inactivation of complement by heat-treatment eliminated the bactericidal activity of plasma against the peptidomimetic-resistant isolates. We were also interested in investigating the activity of peptidomimetic 1 towards the resistant isolates in the presence of plasma. Interestingly, peptidomimetic resistance of the two isolates 2-7 and 7-3 was abolished in the presence of 25% plasma (Table 3), suggesting that the activity of plasma components renders the resistance mechanism against peptidomimetic activity ineffective or even abolishes it completely. Plasma potentiation against resistant strains has been demonstrated for antibiotics [53]. An explanation might be that the resistance mechanisms involve modifications in the outer cell layer, which confers peptidomimetic resistance, but also enhances susceptibility to plasma components. However, plasma potentiation of the activity of peptidomimetics is most likely due to synergy as previously reported [54] as the wild-type strain also exhibited a diminished MIC value when tested in the presence of plasma.

Whole-genome sequencing of twenty selected isolates representing four resistant lineages revealed several mutations that were conserved between lineages, but also mutations that were conserved within lineages indicating that these isolates may be clonal (Table 5). Most of these mutations were found to be related to modification of the cell envelope, which apparently did not seem to entail a fitness cost in resistant isolates since the growth rates of the majority of isolates were comparable to that of the ancestral wild-type strain. No mutations were found in genes encoding known two-component systems. Surprisingly, four mutations had occurred in all ten sequenced isolates originating from lineages no. 4 and 5, which all displayed wild type MIC or low levels of resistance towards peptidomimetic 1 even though the two lineages had a very high population MIC. However, none of these isolates had a MIC exceeding that of the wild-type strain, which may possibly be due to a very low frequency of resistance-conferring mutations in these two lineages. For isolates from lineages no. 2 and 7 two patterns of mutations could be distinguished (Table 5). Thus, the five isolates from lineage no. 2 that were all highly resistant, as well as a highly resistant isolate from lineage no.7 (i.e. 7-7), all shared the same three SNPs and one DIP. Though the presence of the same mutations in lineage no. 2 indicate that these may be clonal, one of these isolates also had two additional mutations, and in addition it is very interesting that these four common mutations were also found in an isolate (7–7) originating from another lineage. One of the four mutations is a SNP in the gene encoding the MltD protein, a membrane-bound lytic murein transglycosylase responsible for peptidoglycan reorganization. This SNP causes a change in amino acid residue 55 of the protein from the nonpolar proline to the polar glutamine, and it is conceivable that this may give rise to changes in the secondary structure, and thereby in the activity of MltD. This is corroborated by a study in *Vibrio anguillarum* where it was shown that inactivation of the *mltD* gene results in resistance to conventional antibiotics [55]. The remaining four isolates from lineage no. 7, of which isolates 7-3 and 7-4 can be considered highly resistant, all displayed the same alternative mutations (Table 5). Hence, it is likely that resistance in these two isolates is due to unidentified mutations in non-coding regions since these isolates appear to be as stable as the population they originate from and therefore their resistance is not likely to be due to population-based resistance mechanisms e.g. epigenetic inheritance [42].

In conclusion, *E. coli* was found capable of developing heritable resistance to an α-peptide/β-peptoid peptidomimetic, and this constitutes the first example where this has been demonstrated for a backbone-modified AMP analogue. Importantly, the acquisition of bacterial resistance towards this type of peptidomimetic did not give rise to cross-resistance to AMPs and did not impair the innate immune system to eliminate the resistant isolates, regardless of which mutations had been induced in the resistant isolates. Thus, in the present study resistance appears to be directly linked to the unnatural scaffold and sequence design displaying alternating cationic and hydrophobic residues. The fact that the developed resistance came with a concomitant increased susceptibility towards soluble plasma constituents (most likely complement) indicate that these peptidomimetics indeed may have a potential as future therapeutics. In addition, this study confirm our previous finding that the present peptidomimetics interact synergistically with plasma [54] as killing of all resistant mutants under these conditions were potentiated to such a degree that the MICs of peptidomimetic 1 was similar to or lower than that of the archetype wild-type ancestor.

However, the finding that resistance in fact may be developed towards peptidomimetics *in vitro* unequivocally show that the resistance issue should be investigated for all types of compounds presently undergoing development similarly to how potential immuno-modulatory properties are already taken into consideration today.

## Materials and Methods

### Bacterial Strain and Culture Conditions

All experiments were performed with *Escherichia coli* ATCC 25922. Stock cultures of the wild-type strain and lineage isolates were stored at –80°C in 4% (w/v) glycerol, 0.5% (w/v) glucose, 2% (w/v) skimmed milk powder and 3% (w/v) tryptone soy powder. Lineage populations were frozen at –80°C in 50% (w/v) glycerol. All experiments were performed at 37°C. Experiments were carried out in cation-adjusted Mueller Hinton II broth (MHB) (Becton Dickinson 212322) adjusted to pH 7.4 or in 1% (w/v) peptone (Becton Dickinson 211677) for MIC determination of natural AMPs. Brain Heart Infusion (BHI) (CM1135) with agar (VWR 20768.292) 1.5% as gelling agent was used throughout for colony plating.

### Antibiotics and Synthesis of Peptidomimetics and Natural AMPs

Polymyxin B (P4932), protamine (P4020), gentamicin (G3632), ampicillin (A9518) and erythromycin (E6376) were purchased from Sigma Aldrich. The α-peptide/β-peptoid peptidomimetics 1, 2 and 3 consisting of alternating repeats of cationic natural α-L-amino acids and unnatural lipophilic β-peptoid residues (Figure 1) were synthesized by solid-phase synthesis as previously described [32], [33]. KR-12 [56], IsCTp [16], PEP-1-K [15] and melittin were prepared by automated microwave (MW)-assisted solid-phase Fmoc-based synthesis on a CEM Liberty microwave peptide synthesizer using a Rink amide resin (loading: 1.0 mmol/g). Fmoc deprotection was performed with 20% piperidine-DMF at 75°C (30 sec followed by 180 sec), while coupling was performed by using the appropriate Fmoc-protected building block (5.0 eq) with DIC (5 eq.) and HOBt (5 eq.) in DMF at 75°C for 15 min. Capping was applied after every fourth coupling with Ac_2_O-DIPEA-NMP (1∶2∶3) at 65°C (30 sec, repeated once). Final deprotection of the N-terminus was followed by cleavage and simultaneously side chain deprotection with TFA-TIS-H_2_O (95∶2.5∶2.5; 3 mL) for 60 min. The filtrate was collected and the resin was eluted with DCM (2 mL) and TFA (2× 2 mL). The combined filtrates were conc. *in vacuo*, and then co-evaporated with toluene (3×). The crude product was triturated with Et_2_O, dissolved in MeCN-H_2_O (50∶50) containing 0.1% TFA, and then purified by preparative HPLC. Finally the product was dissolved in water (1 mL) and lyophilized. Analytical HPLC was carried out on a Phenomenex Luna C18 (2) (3 µm) column (150×4.60 mm) using binary mixtures of eluent A (H_2_O-MeCN-TFA 95∶5∶0.1) and eluent B (H_2_O- MeCN-TFA 5∶95∶0.1) for elution with a flow rate of 0.8 mL/min by using a linear gradient of 10–60% B during 30 min. Peptides were detected with UV at λ = 220 nm. Preparative HPLC was performed on a Luna C18 (2) (5 µm) column (250×21.20 mm) with an Agilent 1100 LC system with a multiple-wavelength UV detector. Elution was performed with a linear gradient of 10–40% during 20 min at a flow rate of 20 mL/min. Peptides were detected with UV at λ = 220 nm. LC-HRMS was performed with a Phenomenex Luna C18 (2) (3 µm) column (150×4.6 mm) using binary mixtures of eluent C (H_2_O-MeCN-HCOOH 95∶5∶0.1) and D (H_2_O-MeCN-HCOOH 5∶95∶0.1). Elution was performed with a linear gradient of 10–60% D during 30 min at a flow rate of 0.5 mL/min. HRMS spectra (ΔM <5 ppm) were obtained using a Bruker MicrOTOF-Q II Quadropol MS detector. Analyt. HPLC (>97% purity at 220 nm) retention times (RT): RT = 16.85 min for KR-12; RT = 28.75 min for mellitin; RT = 17.74 min for IsCTp; RT = 17.79 min for Pep-1-K.

Peptidomimetics and AMPs were solubilised to a stock of 10 mg/mL in sterile MilliQ water and stored at −20°C. Polymyxin B was solubilised to a stock of 10 mg/mL in sterile MilliQ water, filter-sterilized and stored at 5°C. Protamine was solubilised to a concentration of 1024 µg/mL in sterile MilliQ water and used immediately. Gentamicin and ampicillin were solubilised to a stock of 25 mg/mL in sterile MilliQ water, filter-sterilized and stored at 5°C and −80°C, respectively. Erythromycin was solubilised to a stock of 10 mg/mL in 96% ethanol and prepared fresh for each experiment.

### Continuous Selection Experiment

Continuous selection of resistance was performed for peptidomimetic 1 (Figure 1) and for the clinically relevant natural antimicrobial peptide, polymyxin B. A bacterial suspension of a single colony of *E. coli* ATCC 25922 was re-inoculated (10 µl) five times in unsupplemented MHB (990 µl). It was then re-inoculated in MHB supplemented with peptidomimetic 1 (ten lineages) or polymyxin B (ten lineages), and after ten re-inoculations at constant peptidomimetic or polymyxin B concentration, the concentrations were doubled. The starting concentration was 1/16 of the Minimum Inhibitory Concentration, MIC (i.e. 0.5 µg/mL and 0.0625 µg/mL, respectively), and this was increased to 32×MIC during the course of the experiment. Four lineages grown in MHB with MilliQ water were included as control. Re-inoculations were performed twice a day, but were reduced to once a day when growth was slower as assessed by turbidity of the cultures. The total number of passages (supplemented with peptidomimetic/AMP) was 77 times equivalent to approx. 500 generations. For each increase in concentration the lineages were preserved as freezing stocks. Ten colony isolates were randomly selected from each lineage population at 32×MIC and preserved as freezing stocks.

### Determination of MIC

MIC was determined by using the micro-dilution method according to guidelines of the Clinical and Laboratory Standards Institute [57]. Peptidomimetic 1 and erythromycin 1∶2 serial dilutions were prepared from 1024 µg/mL stock solutions to give a final range of 512-0.5 µg/mL in the wells. Ampicillin 1∶2 serial dilutions were prepared from a 256 µg/mL stock solution to give a final range of 128-0.13 µg/mL in the wells. Polymyxin B and gentamicin 1∶2 serial dilutions were prepared from a 64 µg/mL stock solution to give a final range of 32-0.03 µg/mL in the wells. Also, 1∶2 serial dilutions of the AMPs KR-12, IsCTp, melittin and PEP-1-K were prepared from 64 µg/mL stock solutions to give a final range of 32-0.03 µg/mL in the wells; however this was performed in 1% peptone instead of in MHB since activity was drastically diminished in the latter (not shown).

The population lineages were tested for their susceptibility towards peptidomimetic 1, polymyxin B, gentamicin and ampicillin. Single-culture isolates selected from these populations were additionally tested against peptidomimetic 1, polymyxin B, erythromycin and the five reference AMPs. To estimate the level of cross-resistance to closely related peptidomimetics, MIC determinations were performed for peptidomimetics 2 and 3 (Figure 1) towards selected isolates. The bacterial suspensions were grown overnight in MHB at 37°C for determination of the population MIC, or individual isolates were grown overnight on non-selective BHI agar at 37°C. MIC determination performed on the wild-type strain using re-suspended colonies, as well as on a culture grown overnight in broth, showed that pre-growth in broth had minimal effect on the MIC results. In order to determine the population MIC, the respective compound (peptidomimetic 1 or polymyxin B) was added to give a concentration corresponding to 32×MIC to maintain the selection pressure of the freezer-revived lineages, and then MIC determination was performed on the outgrown population (i.e. ∼10^9^ CFU/mL). In all experiments, bacterial cells were suspended in 0.9% saline to give a turbidity of 0.13 at OD_546_ (approximately 1×10^8^ CFU/mL) and diluted in MHB pH 7.4 to a final concentration of 5×10^5^ CFU/mL in each well. Polypropylene plates (Nunc 442587) were used to minimize peptide binding, and the incubation time was 18–20 hours at 37°C. Incubation time was additionally extended up to 44 hours for the individual isolates due to a potential effect of growth rate on the outcome, but no effect was seen of this (not shown). MIC was found in a minimum of two technical (population MIC) or biological (isolate MIC) replicates as the lowest concentration of the AMP or antibiotic where no visible growth was observed.

### Effect of Human Blood Plasma on Growth and Susceptibility

Four isolates, i.e. 2-7, 4-4, 5-6 and 7-3, a representative isolate from four different lineages, were revived from freeze storage. The four isolates were grown in 25–50% blood plasma and in 25–50% heat-inactivated plasma as previously described [54]. Blood plasma was obtained from two different blood donors using sodium citrate as anticoagulant and experiments were performed on at least two independent days. Both donors were fully informed about the study and purpose and a written consent has been received. According to the Danish Science Ethics Committee, the study did not need approval from the Committee (Protocol nr H-2-2013-FSP45). MIC determinations for hydrogen peroxide and lysozyme were performed in biological duplicates as described above to see whether resistance in isolate 2-7 also conferred elevated MIC values towards innate immune factors. Lastly, MIC against peptidomimetic 1 was determined as described above in the presence of 25% plasma (chosen due to lack of growth of several isolates in 50% plasma) to determine whether this would decrease or abolish the elevated MICs of resistant isolates. For this the 1∶2 serial dilutions were made in MHB with 50% blood plasma added to give a final concentration of 25% plasma in the wells.

### Stability of Resistance

Frozen stocks were reconditioned in unsupplemented MHB media for five transfers (∼35 generations) at 37°C. The heritability of resistance was established through MIC determination of bacterial populations as described above.

### Fitness Cost

To evaluate whether resistance altered the physiology of the isolates, we determined the growth rate of all forty isolates from lineage 2, 4, 5 and 7 as well as of *E. coli* ATCC 25922 in unsupplemented MHB media. Additionally, we determined the growth rate of the four isolates 2-7, 4-4, 5-6 and 7-3, in MHB supplemented with peptidomimetic 1 at ½×wild type MIC or 1×wild type MIC (i.e. 4 and 8 µg/mL). To correct for the higher MIC values of isolates 2-7 and 7-3 (which were 16×above the wild type MIC) we also determined the growth rate for *E. coli* ATCC 25922 in MHB supplemented with 1/32 and 1/16×wild type MIC. Optical density readings were obtained at 570 nm at 10–12 minute increments on cultures grown at 37°C over 24 hours (unsupplemented MHB) or 72 hours (supplemented MHB) using the automated Bioscreen C system (Labsystems, Helsinki, Finland). Growth rates were found using linear regression on ln(initial CFU/mL) vs. detection time (i.e. the time until a 0.5 unit increase in absorbance was reached) for inoculum sizes ranging from 10^2^–10^7^ CFU/mL [58]. Data were analysed in GraphPad Prism version 4.03 using One-Way ANOVA followed by Dunnett’s Test to correct for multiple testing. Control groups were *E. coli* ATCC 25922 (in the absence of peptidomimetic) or *E. coli* ATCC 25922/isolates grown in unsupplemented media (in the presence of peptidomimetic). Significance levels were in all tests set at p<0.05. All experiments were performed in duplicate on at least two independent days.

### Whole-genome Sequencing

A total of twenty isolates from four peptidomimetic-exposed lineages were chosen for whole-genome sequencing (Table 4). The isolates were either high-level resistant or low-level resistant or corresponded to wild type MIC. For comparison three isolates from the controls were included. The genome sequence of the ancestral wild type *E. coli* ATCC 25922 has not previously been published and was therefore included as reference. This Whole Genome Shotgun project has been deposited at DDBJ/EMBL/GenBank under the accession number ASHD00000000. The version described in this paper is version ASHD01000000.

Genomic DNA was extracted from each sample by using phenol:chloroform:isoamyl alcohol and then precipitated with isopropanol. Samples were treated with RNAse before quantification and quality analysis using 1% agarose gel electrophoresis, NanoDrop Spectrophotometer (Saveen Werner, Sweden) and Qubit 2.0 Analyser (Invitrogen, United Kingdom). Libraries of 500 bp were used for 100 bp paired-end sequencing of genomes using the Illumina sequencing technology on a HiSeq2000 with a minimum coverage of 100 (Beijing Genomics Institute, Hong Kong, China). *E. coli* ATCC 25922 was *de novo* assembled into contigs using the CLCbio Genomics Workbench (Aarhus, Denmark) resulting in 135 contigs comprising 5,116,439 bp. Using this procedure the 23 isolates were mapped with the 135 contigs as a reference with a minimum coverage of 100, and then single-nucleotide-polymorphism (SNPs) and deletion-insertion-polymorphism (DIPs) with a frequency above 80% were detected.
